# Peripheral and brainstem auditory evaluation in post-COVID-19 individuals

**DOI:** 10.1016/j.clinsp.2024.100472

**Published:** 2024-08-03

**Authors:** Lucas Pinto Mielle, Maria Vanderléia Araujo Maximiano, Ivone Ferreira Neves-Lobo, Liliane Aparecida Fagundes Silva, Alessandra C. Goulart, Carla Romagnolli, Gerson Sobrinho Salvador de Oliveira, Alessandra Giannella Samelli, Carla Gentile Matas

**Affiliations:** a Department of Physical Therapy, Speech-language Pathology and Audiology, and Occupational Therapy, Faculdade de Medicina, Universidade de São Paulo (FMUSP), São Paulo, SP, Brazil; b Department of Epidemiology, School of Public Health, Universidade de São Paulo (USP), São Paulo, SP, Brazil; c Center for Clinical and Epidemiological Research, Hospital Universitário, Universidade de São Paulo (USP), São Paulo, SP, Brazil; d Division of Internal Medicine, Hospital Universitário, Universidade de São Paulo (USP), São Paulo, SP, Brazil; e Infection Control Department, Hospital das Clínicas, Universidade de São Paulo (USP), São Paulo, SP, Brazil

**Keywords:** Audiology, Electrophysiology, Evoked Potentials, Auditory, COVID-19

## Abstract

**Purpose:**

The purpose of this study was to investigate the peripheral and central auditory pathways in adult individuals after COVID-19 infection.

**Method:**

A total of 44 individuals aged between 19 and 58 years, of both genders, post-COVID-19 infection, confirmed by serological tests, with no previous hearing complaints and no risk factors for hearing loss, were assessed. All the participants underwent the following procedures: pure tone audiometry, logoaudiometry, immitanciometry, and Brainstem Auditory Evoked Potentials (BAEP), in addition to answering a questionnaire about auditory symptoms.

**Results:**

Thirteen individuals (29.5 %) had some hearing threshold impairment, mainly sensorineural hearing loss. In the BAEP, 18 individuals (40.9 %) presented longer latencies, mainly in waves III and V. According to the questionnaire answers, 3 individuals (9.1 %) reported worsened hearing and 7 (15.9 %) tinnitus that emerged after the infection. As for the use of ototoxic drugs during treatment, 7 individuals (15.9 %) reported their use, of which 5 showed abnormalities in peripheral and/or central auditory assessments.

**Conclusion:**

Considering the self-reported hearing complaints after COVID-19 infection and the high rate of abnormalities found in both peripheral and central audiological assessments, it is suggested that the new COVID-19 may compromise the auditory system. Due to the many variables involved in this study, the results should be considered with caution. However, it is essential that audiological evaluations are carried out on post-COVID-19 patients in order to assess the effects of the infection in the short, medium, and long term. Future longitudinal investigations are important for a better understanding of the auditory consequences of COVID-19.

## Highlights

• Vestibulocochlear disorders can be caused by COVID-19 infection.
• Sensorineural hearing loss can be present without clinical symptoms.
• Electrophysiological tests suggest an abnormal auditory pathway after COVID-19.
• The use of ototoxic drugs during COVID-19 can worsen hearing.

## Introduction

In 2019, with an abrupt increase in the number of pneumonia cases in the Hubei province of China, a new variant of COVID-19 caused by the infection of the SARS-CoV-2 was identified, and within a few months, it had spread throughout Asian and European countries. On March 11, 2020, the World Health Organization (WHO) declared the new COVID-19 a pandemic.^1^

According to data provided by the WHO, up to April 2024, there were more than 775 million people worldwide with confirmed cases of COVID-19 and the number of deaths has already exceeded 7 million, with more than 38 million confirmed cases and more than 711,000 deaths recorded in Brazil.

It's worth noting that even though it's a respiratory-based disease, there are not only major functional and structural impacts on the respiratory system but also implications for various other systems, the most prevalent of which are neurological and cardiovascular complications, and psychiatric and psychological disorders.^2^

Since the beginning of the COVID-19 pandemic, not only the acute effects have been observed, but also the sequelae caused by the disease. Currently, there has been a significant change in the focus of international health research, from an eminent need to understand the acute symptoms and their treatments, to a more in-depth study of the long-term sequelae from the disease.^2^, ^3^, ^4^, ^5^

More recently, the term “long COVID” has been widely discussed in the literature, characterized by the maintenance of symptoms and alterations even after treatment of the infection and its respiratory symptoms. The mechanisms by which these symptoms are maintained are not yet well known but may be related to dysfunctions caused by the infection, inflammatory processes derived from the immune system, or viral reserves still present in the body.^6^

One of the possible sequelae associated with COVID-19 infection, which has already been described in previous studies with a high number of complaints, is the vestibulocochlear disorders.^7^, ^8^, ^9^ The relationship between airway infections and auditory disorders is already well known, and the correlation between COVID-19 infection and auditory disorders has been under investigation in the past few years.^7^, ^8^, ^9^

Infectious, viral, bacterial, or fungal factors are some of the main causes of temporary or permanent hearing loss, which can affect structures in the outer, middle and inner ear. Moreover, research has shown that viral infections are responsible for approximately 3 % of sudden sensorineural hearing losses among adults.^10^

Wagatsuma et al. analyzed the incidence of hearing loss cases in a population of 2,367 individuals in Japan, between the years 2019 (pre-pandemic) and 2020, and a significant difference was observed, with a 9.5 % incidence rate in 2019 and a 13.2 % incidence rate of hearing loss in 2020, affecting not only the older population (> 60 years), but also the younger population (< 40-years).^11^

With regard to the auditory system, few studies have been carried out and abnormalities in hearing thresholds and Brainstem Auditory Evoked Potentials (BAEP) have been observed in individuals after COVID-19 infection.^12^

Öztürk et al. (2022) compared healthy and post-COVID-19 individuals, through Pure Tone Audiometry (PTA), high-frequency audiometry, Otoacoustic Emissions (OAE), and BAEP, and found statistically significant differences in hearing thresholds at frequencies above 6 kHz, in the OAE amplitudes, and in the absolute latencies of BAEP´s waves I, III and V. The authors concluded that the results suggested direct damage to the outer hair cells in the basal regions of the cochlea, as well as damage to the brainstem due to hypercoagulation.^12^

Bento et al. (2022) carried out a literature review searching for evidence associating COVID-19 and otological symptoms, and found associations between the infection and sudden hearing loss, worse hearing performance after cochlear implant surgery, and abnormal otoacoustic emissions in newborns exposed to the virus during the intrauterine period; they also observed complaints of worsening or triggering of hearing loss, dizziness and tinnitus. The authors concluded that the most probable mechanisms involved would be the inflammation of the cochlea and/or auditory nerve, or ischemic and autoimmune processes.^13^

The direct relationship between COVID-19 infection and hearing loss is still unclear, and even with the currently published data that tends to point to this relationship, proving it is still a difficult and audacious task.^14^, ^15^, ^16^, ^17^, ^18^

Considering the importance of this topic and the low amount of data currently available, the aim of this study was to investigate the peripheral and central auditory pathway in adults after COVID-19, who presented no other risk factors for hearing disorders and no complaints of hearing symptoms before infection.

## Materials and methods

Before any procedure was carried out, all participants were given the opportunity to read and receive the necessary instructions so that they could agree and sign the Informed Consent Form. This cross-sectional clinical study was approved by the institution's Ethics Committee under the number 50778121.0.0000.0068.

The following inclusion criteria were adopted: adult individuals, aged between 18 and 60, of both genders, with a positive diagnosis of COVID-19, who were no longer at risk of transmission and in good health.

The exclusion criteria adopted were a history of hearing loss prior to COVID-19, identified through audiological complaints or audiological tests; occupational noise exposure; continuous use of drugs considered ototoxic or neurotoxic prior to COVID-19 (chemotherapy, aminoglycoside antibiotics, anti-inflammatory drugs-acetylsalicylic acid and others),^19^ and otologic surgeries.

Due to the necessity to exclude other factors that could be involved in hearing disorders, the absence of hearing complaints prior to the COVID-19 infection proved indispensable. Due to a lack of self-demand of audiological assessments in this group, no individual was found that had been evaluated by audiological exams prior to the infection. Therefore, to minimize the possibility of influences of other factors in the results, an audiological questionnaire was applied and a rigorous exclusion criteria was adopted. The anamnesis used to identify possible factors related to hearing loss, contemplated topics such as hypoacusis, tinnitus, dizziness, otalgia, pruritus, ear fullness, hyperacusis, listening behaviors in everyday situations, comorbidities, smoking, alcoholism, exposure to noise and the use of ototoxic drugs.

**Fig. 1:**
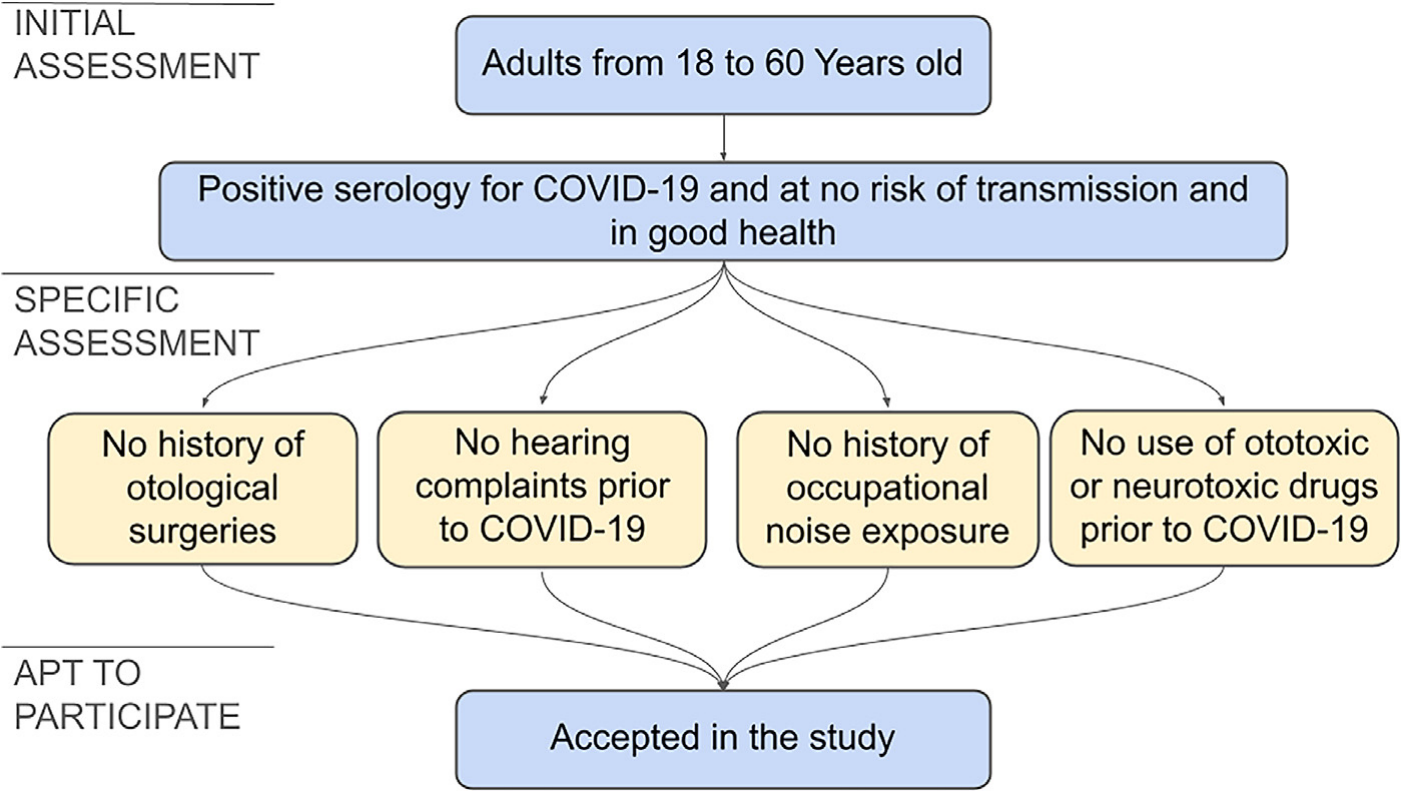
Inclusion and exclusion criteria flowchart.

The selection process of the cohort is shown in Fig. 1.

All participants were evaluated by the following procedures:

1) Clinical anamnesis to obtain information about the participant's medical history, as well as otological complaints, surgeries, occupational noise exposure, or prolonged use of ototoxic medication.
2) Specific questionnaire on COVID-19-related issues related to the number of infections, vaccination history, hospitalization, and specific questions on hearing complaints, such as impaired hearing acuity, tinnitus and dizziness, and other symptoms such as olfactory, cognitive, and psychological disorders.
3) Meatoscopy (Heine Otoscope) to inspect the external acoustic meatus in order to identify any obstructions that could hinder or impede the audiological assessment procedures.
4) Imitanciometry (Interacoustic Imitanciometer, model AT 235), consisting of tympanometry and ipsi- and contralateral acoustic reflexes at 500Hz, 1000Hz, 2000Hz, and 4000 Hz. The normality criteria for tympanometry were based on Jerger (1970),^20^ and the acoustic reflexes were classified in terms of the presence or absence of response (when the amplitude of the reflex was lower than 0.05 mL).
5) Pure Tone Audiometry (PTA) (Interacoustic audiometer, model AC 40), carried out in a soundproof booth (in compliance with ANSI S3.1 ‒ 1991 environmental noise quantity standard), by air conduction, at 250Hz, 500Hz, 1000Hz, 2000Hz, 3000Hz, 4000Hz, 6000Hz and 8000 Hz frequencies and, if necessary (in the presence of thresholds higher than 20 dB/HL), by bone conduction at 500Hz, 1000Hz, 2000Hz and 4000 Hz frequencies. The criteria for classifying the type and degree of hearing loss were in accordance with Lloyd and Kaplan.^21^
6) Logoaudiometry, divided into: the Speech Recognition Threshold (SRT), which had to be compatible with the average of the tonal thresholds at frequencies of 500Hz, 1000Hz and 2000 Hz or up to 10 dB/HL above, and the Percentage Speech Recognition Index (PSRI), which was considered normal if it was above 88 %.^22^
7) Brainstem Auditory Evoked Potentials (BAEP) (two-channel Smart EP USB Jr equipment from Intelligent Hearing Systems (HIS 5020), Miami-Florida), carried out using an ER-3A insert earphone and Ag/AgCI electrodes positioned at Fz (active electrode), Fpz (ground) and on the right (M2) and left (M1) mastoids. The stimulus used was the click, at 80 dBnHL, presenting 2,048 stimuli monaurally, with rarefied polarity and a rate of 27.7 stimuli per second, in two collections aimed at reliable reproducibility. The analysis was carried out in relation to the absolute latency values (ms) of waves I, III and V, and the interpeaks I‒III, III‒V and I‒V, according to the normality standard proposed in the User Manual.^23^

Data analysis initially consisted of calculating the percentage of normal and abnormal results in each procedure. To investigate the association between normal and abnormal PTA and BAEP results with self-reported hearing symptoms after COVID-19 (hypoacusis, dizziness, and tinnitus) and the use of ototoxic drugs, an inferential analysis using the Chi-Square test with continuity correction was carried out.

As for the numerical variables, a descriptive analysis of the data was carried out and the difference between the right and left ears was compared using the unpaired Student's *t*-test.

For all the analyses, *p*-value < 0.05 was considered significant.

## Results

A total of 44 individuals aged between 19 and 58 were evaluated from 6 to 24 months after COVID-19 infection. The sample was composed of individuals who attended the University Hospital of the research institution, as well as by spontaneous demand.

Of the 44 individuals, seven reported the use of ototoxic drugs during the treatment following COVID-19 infection such as antibiotics, anti-inflammatory and diuretics (Table 1).

**Table 1:** Characterization of the sample (*n* = 44).

	Minimum	Maximum	Mean	Standard deviation
Age (years)	19	58	39.7	13.6
	Number of participants		Percentage (%)	
**Gender**				
Female	35		79.5 %	
Male	9		20.5 %	
**Comorbidities**				
Systemic arterial hypertension	4		9.1 %	
Pneumonia	2		4.5 %	
Type II diabetes	1		2.3 %	
High levels of cholesterol	1		2.3 %	
Use of Ototoxic drugs				
Yes	7		15.9 %	
No	37		84.1 %	

Most of the individuals included had only contracted the infection once (90.9 % of cases) and did not require hospitalization (54.5 %) or had only one hospitalization (40.9 %). In addition, at the moment of the assessment, the majority had already received all four doses of an immunizer (52.3 % of cases) (Table 2). The most used vaccine was the AstraZeneca, applied on 20 individuals (45.5 %), followed by Pfizer at 18 individuals (40.9 %), Janssen at three individuals (6.8 %), CoronaVac at two individuals (4.5 %) and Oxford at one individual (2.3 %).

**Table 2:** Characterization of COVID-19 infections (*n* = 44).

	Number of participants	Percentage (%)
**Number of infections**		
1 infection	40	90.9 %
2 infections	3	6.8 %
3 infections	1	2.3 %
**Doses of vaccine**		
1 dose	0	0.0 %
2 doses	2	4.5 %
3 doses	19	43.2 %
4 doses	23	52.3 %
**Number of hospitalizations**		
None	24	54.5 %
1 hospitalization	18	40.9 %
2 hospitalizations	2	4.5 %

As for self-reported vestibulocochlear complaints after infection, dizziness was the main symptom, affecting 12 patients (27.3 % of cases), and this symptom remained even after the infection ceased in four of them. In addition, non-auditory symptoms were also observed, the most frequently being the loss of smell and taste, followed by memory difficulties and fatigue; memory difficulties were the main symptom that persisted after the cessation of the infection (22.7 % of cases) (Table 3).

**Table 3:** Self-reported symptoms after COVID-19 according to questionnaire (*n* = 44).

	Only during infection		During and after infection		Total		p-value
	Number of participants	Percentage (%)	Number of participants	Percentage (%)	Number of participants	Percentage (%)	
**Vestibulocochlear symptoms**							
Hypoacusis	0	0.0 %	3	6.8 %	3	6.8 %	Not available
Tinnitus	3	6.8 %	4	9.1 %	7	15.9 %	0.570
Dizziness	8	18.2 %	4	9.1 %	12	27.3 %	0.323
**Non-auditory symptoms**							
Depression	2	4.5 %	3	6.8 %	5	11.4 %	0.752
Stress and anxiety	4	9.1 %	5	11.4 %	9	20.5 %	0.453
Fatigue	10	22.7 %	3	6.8 %	13	29.5 %	0.331
Memory difficulties	3	6.8 %	10	22.7 %	13	29.5 %	0.331
Loss of smell / taste	12	27.3 %	3	6.8 %	15	34.1 %	0.613

**Fig. 2:**
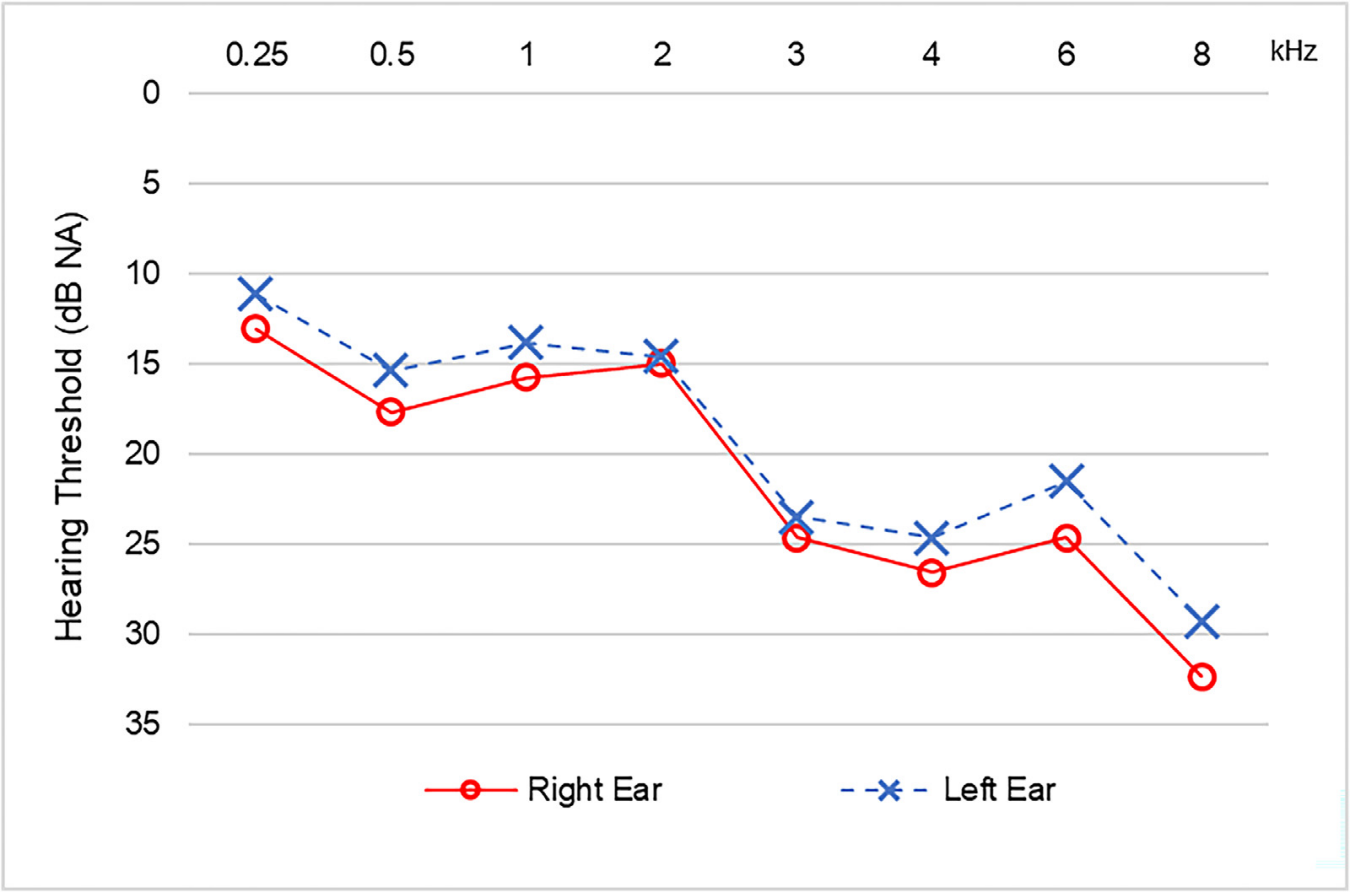
Comparison of the mean values of hearing thresholds by frequency among patients with altered thresholds for each ear (*n* = 12).

The PTA showed 31 patients with normal results bilaterally (70.5 %), two patients with unilateral hearing loss (4.5 %), and 11 patients with bilateral hearing loss (25 %). Among the patients with higher hearing thresholds, most presented mild sensorineural hearing loss. The results for each ear are shown in Table 4, and there was no significant difference in hearing thresholds between the ears (*t* = 2.18; *p*-value > 0.05) (Fig. 2). As far as logoaudiometry is concerned, the results obtained in SRT and PSRI were compatible with PTA.

**Table 4:** PTA results with hearing loss categorized by type and degree for each ear (*n* = 44).

	Right ear		Left ear	
	Number of participants	Percentage (%)	Number of participants	Percentage (%)
**Normal**	33	75.0 %	33	75.0 %
**Mild SNHL**	6	13.6 %	7	15.9 %
**Mild to moderate SNHL**	4	9.1 %	3	6.8 %
**Moderate SNHL**	0	0.0 %	1	2.3 %
**MHL**	1	2.3 %	0	0.0 %

SNHL, Sensorineural Hearing Loss; MHL, Mixed Hearing Loss.

The immitanciometry results showed Type A tympanometric curves in 43 (97.7 %) individuals and Ar in one individual (2.3 %). The acoustic reflexes were compatible with the respective PTA thresholds in all subjects.

There were no significant differences between the ears in BAEP (*p*-value > 0.05) (Table 5). Longer latencies were observed in 18 patients (40.9 %), and the most prevalent abnormalities were in waves III and V (Table 5, Table 6).

**Table 5:** Comparative analysis of absolute and interpeak latencies of BAEP waves for each ear in milliseconds (ms) (*n* = 44).

	Mean (ms)	Standard Deviation	*t*-value	p-value
**Wave I**				
Right Ear	1.67	0.12	2.02	0.186
Left Ear	1.64	0.13		
**Wave III**				
Right Ear	3.82	0.13	2.02	0.132
Left Ear	3.78	0.18		
**Wave V**				
Right Ear	5.68	0.20	2.02	0.104
Left Ear	5.64	0.19		
**Interpeak I‒III**				
Right Ear	2.16	0.14	2.02	0.873
Left Ear	2.15	0.15		
**Interpeak III‒V**				
Right Ear	1.86	0.14	2.02	0.604
Left Ear	1.85	0.16		
**Interpeak I‒V**				
Right Ear	4.02	0.20	2.02	0.579
Left Ear	4.00	0.20

**Table 6:** Qualitative analysis of the abnormalities observed in the BAEP for each ear (*n* = 44).

	Right ear		Left ear	
	Number of participants	Percentage (%)	Number of participants	Percentage (%)
**Wave I**				
Right Ear	38	86.4 %	37	84.1 %
Left Ear	6	13.6 %	7	15.9 %
**Wave III**				
Right Ear	35	79.5 %	34	77.3 %
Left Ear	9	20.5 %	10	22.7 %
**Wave V**				
Right Ear	35	79.5 %	36	81.8 %
Left Ear	9	20.5 %	8	18.2 %
**Interpeak I‒III**				
Right Ear	42	95.5 %	42	95.5 %
Left Ear	2	4.5 %	2	4.5 %
**Interpeak III‒V**				
Right Ear	42	95.5 %	40	90.9 %
Left Ear	2	4.5 %	4	9.1 %
**Interpeak I‒V**				
Right Ear	42	95.5 %	40	90.9 %
Left Ear	2	4.5 %	4	9.1 %

Subsequently, an association analysis was carried out between the self-reported hearing symptoms and the use of ototoxic medication reported in the specific COVID-19 questionnaire and the PTA and BAEP results. There was an association between PTA and hypoacusis, dizziness, and the use of ototoxic drugs (Table 7). However, no association was observed for BAEP (Table 8).

**Table 7:** Association between PTA results and self-reported hearing symptoms and use of ototoxic drugs (*n* = 44).

	Normal PTA		Abnormal PTA		Chi-Square	p-value
	n	%	n	%		
**Current hypoacusis**						
Yes	0	0.0 %	3	6.8 %	8.585	0.003*
No	32	72.7 %	9	20.5 %		
**Tinnitus after COVID-19**						
Yes	4	9.1 %	3	6.8 %	3.745	0.053
No	29	65.9 %	8	18.2 %		
**Dizziness after COVID-19**						
Yes	6	13.6 %	6	13.6 %	4.297	0.038*
No	26	29.1 %	6	13.6 %		
**Ototoxic drug use**						
Yes	2	4.5 %	5	11.4 %	8.183	0.004*
No	30	68.2 %	7	15.9 %		

PTA, Pure Tone audiometry; N, Number of participants; %, Percentage.

**Table 8:** Association between BAEP results and self-reported hearing symptoms and use of ototoxic drugs (*n* = 44).

	Normal BAEP		Abnormal BAEP		Chi-square	p-value
	n	%	n	%		
**Current hypoacusis**						
Yes	1	2.3 %	2	4.5 %	0.884	0.347
No	25	56.8 %	16	36.4 %		
**Tinnitus after COVID-19**						
Yes	3	6.8 %	4	9.1 %	0.908	0.341
No	23	52.3 %	14	31.8 %		
**Dizziness after COVID-19**						
Yes	6	13.6 %	6	13.6 %	0.564	0.453
No	20	45.5 %	12	27.3 %		
**Ototoxic drug use**						
Yes	3	6.8 %	4	9.1 %	0.908	0.341
No	23	52.3 %	14	31.8 %		

BAEP, Brainstem Auditory Evoked Potential; N, Number of participants; %, Percentage.

## Discussion

The aim of this study was to assess the peripheral and central auditory pathways in adults infected by COVID-19. To do this, the authors assessed patients with no other risk factors for hearing loss and no complaints of hearing symptoms prior to infection.

In the sample evaluated, it was observed that the majority of the individuals evaluated were female and that most of them had been infected only once (90.9 %) and 45.5 % needed hospitalization.

Among the symptoms reported by the participants, loss of smell and taste (34.1 %) and fatigue (29.5 %) were the most frequently reported. On the other hand, memory difficulties were the main complaint that emerged at the onset of the infection (29.5 %), and which continued even after the end of the infection (22.7 %). Butowt et al.,^24^ describe a worldwide prevalence of 44.1 % of anosmia and 43.3 % of dysgeusia and propose four hypotheses: obstruction or damage to neural receptors, brain dysfunction, or damage to the cells that support these systems. Boscolo-Rizzo et al.,^25^ investigated complaints of anosmia or dysgeusia in 403 individuals and found a prevalence of 66.3 % in this population soon after infection, and after three years 92.1 % reported total cessation of symptoms; the results found in this study corroborating this finding, with 80 % of individuals who reported complaints of anosmia or dysgeusia showing cessation of symptoms. Another study investigated the prevalence of fatigue in individuals after COVID-19 and found that 32 % of individuals complained of fatigue 12 weeks or more after the infection ended.^26^

As for vestibulocochlear symptoms, the main self-reported complaint was dizziness in 27.3 % of individuals, and this was a symptom that remained even after the cessation of infection in 9.1 % of cases. Aldè et al.^26^ investigated the prevalence of dizziness and vertigo in mild to moderate post-COVID-19 individuals, analyzing a population of 1,512 individuals who attended from October 2020 to March 2021 at an Italian health service, and found that 16.6 % of individuals had dizziness and 12 % vertigo.

The PTA evaluation showed that 13 individuals (29.5 %) had peripheral audiological disorders, most of whom had sensorineural hearing loss. As for the degree of hearing loss, most of the patients presented mild hearing loss, with main abnormalities in the higher frequencies, from above 2 kHz.

These results had already been described by Dusan et al.,^27^ who performed PTA on 74 individuals, using inclusion criteria similar to those of the present study, and found 30 individuals (40.5 %) with sensorineural hearing loss, with major impairment in the high frequencies. Öztürk et al. also observed sensorineural hearing loss, with the main impact at frequencies above 6 kHz. The authors emphasized that viruses from the same family (MERS and SARS) have already been observed interfering with the auditory system, both through cochlear damage directly affecting hair cells, especially those located in the more basal regions of the cochlea (responsible for the higher frequencies), as well as direct damage to the brainstem. The authors also proposed a third hypothesis, that this disorder is the result of hypercoagulation, which is regularly observed in infected patients, and that this coagulation results in cochlear ischemia and, subsequently, cell death.^12^

With regard to the results of the central audiological assessments, the most observed abnormality was increased wave III latency, followed by an increase in wave V latency. This increase in absolute latencies in BAEP corroborates the results of Öztürk et al.^12^ who studied a population of 30 individuals, aged 18 to 45, diagnosed with COVID-19, with no previous hearing complaints, and compared them with a group of 30 individuals with no hearing complaints. High-frequency audiometry, BAEP, and otoacoustic emissions tests were carried out and found a significant difference in thresholds from 4 to 14 kHz between the groups, lower amplitudes in otoacoustic emissions, and longer absolute latencies in BAEP for the study group.

Regarding the presence of complaints of hypoacusis, all the individuals who reported decreased hearing after COVID-19 had hearing loss (6.8 %), and of these, two had abnormal BAEP. However, of those without complaints, nine individuals (20.5 %) also presented hearing loss. Dharmarajan et al.^28^ assessed 100 individuals aged between 21 and 60, with no previous hearing complaints and COVID-19 confirmed, using PTA and otoacoustic emissions, and found 53 individuals (53 %) with sensorineural hearing loss, of whom only 11 (20.7 % of the individuals with abnormalities) reported worsening hearing. These findings reinforce the importance of audiological assessment in post-COVID-19 individuals, even without hearing complaints.

With regard to tinnitus, this was a complaint reported by seven individuals (15.9 %), of whom 3 (6.8 %) had abnormalities in the peripheral assessments and 4 (11.4 %) had abnormalities in the central assessments. In 2022, Meng et al. found several case studies and clinical trials that described complaints of tinnitus and dizziness in this population, the researchers reported that the mechanisms by which these disorders occur are not yet well defined, but these findings are important and similar to those obtained in the present study.^8^

Similarly, the complaint of dizziness was found in 12 individuals, six of whom had hearing loss and BAEP abnormalities. This complaint also seems to be associated with possible disorders of the vestibular system, which has been verified and described by Aldè et al.^26^ and Korres et al.^29^

It is known that the use of ototoxic drugs is a reality in the treatment of COVID-19. In this study, the authors found that seven individuals (15.9 %) had used ototoxic drugs during treatment and, when comparing them with the audiological results, it was noted that of these, 5 (11.4 %) showed abnormalities in the peripheral evaluations and 4 (9.1 %) in the central evaluations. Therefore, one of the hypotheses is that these factors may be related to ototoxic drugs, which can cause damage to the inner ear, such as damage to hair cells of the cochlea.^30^ Therefore, audiological monitoring, as occurs in other cases of long-term use of ototoxic drugs, should be a measure adopted in this population.

The relationship between viral infections and both congenital and acquired hearing disorders is already well-established in the literature. Studies have reported that approximately 3 % of sudden sensorineural hearing loss in adults is due to viral infections,^9^ and approximately 40 % of congenital hearing loss is caused by cytomegalovirus.^31^

The pathophysiology by which the authors explain these auditory disorders is diverse, such as direct damage to cochlear structures such as the hair cells, atrophy of the stria vascularis, fibrosis of the tympanic and vestibular scales, or damage to the organ of Corti itself.^31^ With regard to neural structures, authors cite direct damage to the auditory nerve, as it occurs in cases of rubella, the herpes virus, and mumps. However, other mechanisms that act more indirectly on the auditory system have been hypothesized, such as secondary opportunistic infections in immunosuppressed individuals, which is the case of patients receiving treatment for HIV.^32^ However, some authors have also reported possible indirect damage to the auditory system due to the use of ototoxic drugs in the treatment of certain viral infections or, in some cases, damage to the auditory system due to the action of the individual's immune system in the face of a viral infection.^18^^,^^31^, ^32^, ^33^, ^34^, ^35^, ^36^

Shin et al.^4^ reported that, as this is a neurotropic virus, it has an affinity for invading regions of the nervous system. The authors found that 30 % to 40 % of autopsies carried out on patients after COVID-19 showed viral signs in brain regions. In addition, neurodegenerative alterations were found in the brainstem and its connections. They emphasized that, in some cases, abnormalities in the brainstem have been found in neurodegenerative conditions, even without the presence of viral material, indicating that this damage may be due not only to direct damage to the brainstem but also to alterations secondary to infection, such as vascular alterations, immune responses or inflammation.

Currently, the exact mechanisms that can establish the causal relationship between COVID-19, regarding infection, treatment, and vaccination, and hearing system disorders are still unknown, but the evidence both in the current literature^4^^,^^37^ and in the present study tends to indicate an alteration at both peripheral and central auditory pathways.

This study had limitations in terms of sample size due to low adherence to audiological procedures, as well as the impossibility of performing an intra-individual pre- and post-COVID-19 comparison, due to the difficulty of finding individuals without audiological complaints who had undergone audiological tests prior to COVID-19 infection.

## Conclusion

In this study, post-COVID-19 adults (both with and without post-COVID-19 hearing complaints) showed alterations in the peripheral and central auditory pathways.

Due to the many variables involved in this study, the results should be considered with caution. However, it is essential that audiological assessments are carried out on patients diagnosed with COVID-19 so that the effects of the infection can be assessed in the short, medium, and long term. Future studies, especially longitudinal ones, are of great importance for a better understanding of the auditory effects of COVID-19.

## Authors’ contributions

LPM and MVAM: Literature, data acquisition and analysis, and manuscript preparation and editing.

LAFS: Data analysis, statistical analysis, and manuscript preparation and editing.

IFNL: Design, data analysis, and manuscript preparation and editing.

AGS and CGM: Concepts, design, and manuscript final review.

## Funding

This study was financed in part by the São Paulo Research Foundation, FAPESP ‒ Finance Code 078 ‒ Process number: 2021/11373-9

## Declaration of competing interest

The authors declare no conflicts of interest.
